# Supplementary material for: Complexome profiling of the Chlamydomonas psb28 mutant reveals TEF5 as an early PSII assembly factor
Source: Plant Cell. 2025 Mar 17;37(6):koaf055. doi: 10.1093/plcell/koaf055 (PMC12164590; doi:10.1093/plcell/koaf055)
Supplement: koaf055_Supplementary_Data [file koaf055_supplementary_data.zip › psb28_interactive.zip!/plots/intensity/qualityPlots/peptides/ALD5_Cre12.g500150.t1.1.html]

### Cre12.g500150.t1.1

Trivial name: ALD5  
SeqCoverage [median]: 57.10  
Mass: 57.93 kDa  
  
 

| Sequence | MissedCleavage | Mass [Da] | wtIntensity | muIntensity | Score |
| --- | --- | --- | --- | --- | --- |
| ANNSPYGLAAGVFSNNIDTVNTLTR | 0 | 2608.29 | 1645.85 | 0.00 | 102.95 |
| AVYQPLSNPAWR | 0 | 1400.72 | 1516.40 | 1775.58 | 111.61 |
| EEIFGPVQSIMK | 0 | 1376.70 | 845.12 | 0.00 | 70.03 |
| EKGEYALSNYTQVK | 1 | 1628.80 | 2611.05 | 7735.26 | 148.69 |
| EVGLPDGVLNVVTGYGPTAGNR | 0 | 2184.12 | 1887.95 | 0.00 | 135.23 |
| GEYALSNYTQVK | 0 | 1371.67 | 688.40 | 0.00 | 83.87 |
| GYYVEPTVFADVKDDMK | 1 | 1975.92 | 3034.29 | 4025.39 | 70.78 |
| HVDVPFAIDHLR | 0 | 1417.75 | 4005.94 | 12118.49 | 143.97 |
| IAREEIFGPVQSIMK | 1 | 1716.92 | 0.00 | 3024.12 | 79.02 |
| ILSYIDSGKR | 1 | 1150.63 | 6152.35 | 9289.80 | 77.38 |
| KTMPVVDPR | 1 | 1041.56 | 0.00 | 585.89 | 56.40 |
| KVGDPFSSVEQGPQVDDDQFKK | 2 | 2449.18 | 3189.41 | 4342.42 | 64.30 |
| LADAMEAHVDELAQLETLDNGKPFFYSR | 1 | 3179.52 | 801.23 | 0.00 | 68.07 |
| LGPALAAGNTVVLKPAEQTPMTALK | 1 | 2490.39 | 6071.63 | 18053.93 | 99.08 |
| LPNQLFIDGK | 0 | 1143.63 | 443.32 | 3440.19 | 102.06 |
| RAFDTGPWPR | 1 | 1201.60 | 0.00 | 512.07 | 39.92 |
| SALSYAQK | 0 | 866.45 | 2270.41 | 2158.96 | 86.83 |
| SLDDVIAR | 0 | 887.47 | 1609.47 | 6846.25 | 180.70 |
| TAFTGSTEVGR | 0 | 1124.55 | 2196.79 | 1730.70 | 96.33 |
| TEEVVVEVAEGDAADVDRAVEAAR | 1 | 2499.21 | 1298.82 | 0.00 | 100.67 |
| TEEVVVEVAEGDAADVDRAVEAARR | 2 | 2655.31 | 733.05 | 0.00 | 24.55 |
| VASHPGVDKTAFTGSTEVGR | 1 | 2015.01 | 4905.48 | 0.00 | 64.68 |
| VGDPFSSVEQGPQVDDDQFKK | 1 | 2321.08 | 3336.17 | 7409.17 | 152.01 |
| VYVHEAVYDEFVR | 0 | 1624.79 | 2483.78 | 8177.07 | 119.39 |
| VYVHEAVYDEFVRK | 1 | 1752.88 | 967.72 | 1126.17 | 65.47 |
|  |  |  | 52694.6 | 92351.5 |  |
